# Insights from tissue-specific transcriptome sequencing analysis of *Triatoma infestans*

**DOI:** 10.1590/0074-02760160473

**Published:** 2017-06

**Authors:** Leilane O Gonçalves, de Oliveira Luciana M, C D’Ávila Pessoa Grasielle, Aline CL Rosa, Marinely G Bustamante, Carlota J Belisário, Daniela M Resende, Lileia G Diotaiuti, Jeronimo C Ruiz

**Affiliations:** 1 Fundação Oswaldo CruzFundação Oswaldo CruzCentro de Pesquisas René RachouGrupo Informática de Biossistemas e GenômicaBelo HorizonteMGBrasilFundação Oswaldo Cruz-Fiocruz, Centro de Pesquisas René Rachou, Grupo Informática de Biossistemas e Genômica, Belo Horizonte, MG, Brasil; 2 Fundação Oswaldo CruzFundação Oswaldo CruzCentro de Pesquisas René RachouBelo HorizonteMGBrasilFundação Oswaldo Cruz-Fiocruz, Centro de Pesquisas René Rachou, Programa de Pós-Graduação em Ciências da Saúde, Belo Horizonte, MG, Brasil; 3 Universidade Federal de Minas GeraisUniversidade Federal de Minas GeraisInstituto de Ciências BiológicasBelo HorizonteMGBrasilUniversidade Federal de Minas Gerais, Instituto de Ciências Biológicas, Programa de Pós-Graduação em Bioinformática, Belo Horizonte, MG, Brasil; 4 Fundação Oswaldo CruzFundação Oswaldo CruzCentro de Pesquisas René RachouLaboratório de Triatomíneos e Epidemiologia da Doença de ChagasBelo HorizonteMGBrasilFundação Oswaldo Cruz-Fiocruz, Centro de Pesquisas René Rachou, Laboratório de Triatomíneos e Epidemiologia da Doença de Chagas, Belo Horizonte, MG, Brasil; 5 Fundação Oswaldo CruzFundação Oswaldo CruzInstituto Oswaldo CruzRio de JaneiroRJBrasilFundação Oswaldo Cruz-Fiocruz, Instituto Oswaldo Cruz, Programa de Pós-Graduação em Biologia Computacional e Sistemas, Rio de Janeiro, RJ, Brasil

**Keywords:** Next Generation Sequencing, Triatoma infestans, transcriptome assembly

## Abstract

*Triatoma infestans* is an insect of subfamily Triatominae (Hemiptera: Reduviidae) and an important vector of *Trypanosoma cruzi*, the etiologic agent of human Chagas disease. In this work we reported a transcriptome assembly and annotation of *T. infestans* heads obtained by Next Generation Sequencing (NGS) technologies.

*Triatoma infestans* is one of the vectors of *Trypanosoma cruzi*, the etiologic agent of human Chagas disease in South America. It’s estimated that about 6-8 million people, mostly in Latin America, are infected worldwide and approximately 10,000 deaths per year were reported (Medone et al. 2015). *T. infestans* has high anthropophily, high colonisation capacity and high rates of infection. Because of these features, this triatominae is the main vector in the countries where it occurs, Bolivia, Argentina and Paraguay (Silveira 2002, Coura & Dias 2009), where *T. infestans* populations that are resistant to pyrethroid insecticides have been a problem for the Chagas disease control (Vassena & Picollo 2003, Audino et al. 2004, Picollo et al. 2005, Toloza et al. 2008, Germano et al. 2010, Lardeux et al. 2010, Gomez et al. 2015). In order to obtain the transcriptional profile of *T. infestans*, a total of seven samples from two Bolivian populations (Chaco and Bolivian Valley) were used. Each sample was a pool of twenty heads from nymphs on the third instar and was sequenced separately. Heads were chosen with the intention of obtaining transcripts that could be related to the neurotoxicity of the insecticides. The RNA was extracted using TRIzol® Reagent kit (Invitrogen) and measured in Nanodrop® (Invitrogen). To the cDNA synthesis it was used 50 µg of RNA. The sequencing of the paired end mRNA enriched libraries was performed on Illumina MiSeq platform. A total of 11,731,170 reads were generated, and the quality control was performed with PRINSEQ (Schmieder & Edwards 2011). Data filtering and trimming was performed with Trimmomatic (Bolger et al. 2014). Sequence artifacts such as sequencing adapters were removed using data available at Trimmomatic software package. Using a cutoff of Phred quality ≥ 25 and a minimum read length of 100 bp, a total of 10,335,132 reads from all samples were assembled together using Trinity (Haas et al. 2013) and for reduce the transcripts redundancy Cap3 (Huang & Madan 1999) was also applied. The detailed information about *de novo* transcriptome assembly is reported in Table. The Trinity package identified a total of 65,043 transcripts with a median length of 313 bp and a N_50_ value of 466.

**TABLE:** Summary of transcriptome assembly

Total of Trinity ‘genes’	62,449	
Total of Trinity transcripts	65,043	
G + C content	36,59%	
N_50_	466	
Median contig length	313 bp	
Total of Cap3 contigs	5,219
Total of Cap3 singlets	50,680	

The functional annotation was performed using BLAST (Altschul et al. 1990) searching tool against non-redundant (NR) protein from NCBI and Swiss-Prot databases. The functional annotation results revealed that 58% and 72% of the proteins presented no hits to known sequences in the NR and Swiss-Prot databases, respectively. Regarding the NR comparison, among the proteins assigned with biological functions, ~38% (9,111 proteins) have high sequence similarity (≥ 70%) with *Cimex lectularius* and ~23% (5,390 proteins) with *Halyomorpha halys*. Only 275 (~1%) proteins could be assigned for *Triatoma* spp and 163 (~0.7%) proteins to *Rhodnius prolixus*. None of the transcripts obtained was related to the cytotoxicity of pyrethroid insecticides.

Summarising, this work has the potential to contribute to a better understanding of the transcriptional profile linked with one of the most important vectors of the etiologic agent of human Chagas disease, *T. infestans*.

*Accession codes* - The BioProject accession number is PRJNA348445. The data used in this project has been deposited at SRA under the accession numbers SRR4427078, SRR4427079, SRR4449814, SRR4449815, SRR4449939, SRR4449940, SRR4449941 and BioSamples codes: SAMN05908558 and SAMN05908559.
